# Identifying early warning signals of cancer formation

**DOI:** 10.1002/qub2.81

**Published:** 2025-01-23

**Authors:** Chong Yu, Wenbo Li, Xiaona Fang, Jin Wang

**Affiliations:** ^1^ State Key Laboratory of Electroanalytical Chemistry Changchun Institute of Applied Chemistry Chinese Academy of Sciences Changchun China; ^2^ Department of Statistics Jilin University of Finance and Economics Changchun China; ^3^ Department of Chemistry Northeast Normal University Changchun China; ^4^ Joint Research Centre on Medicine the Affiliated Xiangshan Hospital of Wenzhou Medical University Ningbo China; ^5^ Center for Theoretical Interdisciplinary Sciences Wenzhou Institute University of Chinese Academy of Sciences Wenzhou China; ^6^ Department of Chemistry and of Physics and Astronomy State University of New York at Stony Brook Stony Brook New York USA

**Keywords:** cancer early warning signals, gene regulatory network, landscape, flux, entropy production rate, time irreversibility

## Abstract

It is increasingly clear that cancer is a complex systemic disease and one of the most fatal diseases in humans. Complex systems, including cancer, exhibit critical transitions in which the system abruptly shifts from one state to another. However, predicting these critical transitions is difficult as the system may show little change before the tipping point is reached. Models for predicting cancer are generally not accurate enough to reliably predict where these critical transitions will occur. Additionally, there is often a gap between theoretical results and clinical practice. To address these issues, we conducted a study using gastric cancer as a representative to reveal the tipping point of cancer and develop a feasible method for clinical monitoring. We used gene regulatory networks and a landscape framework to quantify the formation of gastric cancer. Since the dissipation cost of cancer cells is different from that of normal cells, we calculated the entropy product rate (EPR) and mean flux to quantify the thermodynamic cost and dynamical driving force in predicting critical transitions of cancer, which can serve as early warning signals. Both the EPR and mean flux change sharply near the point when the cancer state is about to emerge and/or the normal state is about to disappear. Moreover, the peak or sharp upward trends of the signals occur much earlier than critical slowdown and flickering frequency. These significant variations can be used as early warning signals for cancer. To further explore early warning signals in clinical and experimental trials, we calculated the difference in cross correlations (Δ*C*) forward and backward in time for the stochastic gene expression time series. This time‐irreversible measure gives a rise to peak before the bifurcation points, which can help detect precancerous and metastatic early warning signals in clinical practice rather than just theoretical calculation. This study is crucial for effectively identifying early warning signals for cancer in clinical and experimental settings.

## 1 INTRODUCTION

Cancer is one of the deadliest diseases worldwide, and despite tremendous efforts in cancer research, progress has been limited. Early diagnosis is critical for postoperative survival and effective cancer treatment. Gastric cancer is a prime example: its survival rate is poor globally, with a 5‐year survival rate of only around 10%. However, in developed countries such as Japan, where early diagnosis rates are around 50%, the 5‐year survival rate can reach up to 90% [1]. Therefore, detecting methods that enable the diagnosis of cancer at an early stage are crucial for the effective cancer treatment.

It is now widely recognized that many complex systems have critical thresholds beyond which the system switches abruptly from one state to another. Cancer is a complex systemic disease, and these critical thresholds can be seen as the tipping points when the disease progresses to a cancerous state. Detecting these critical thresholds is of great significance for early warning of cancer. However, predicting the critical transitions is challenging because the state of the system may show little change before the tipping point is reached. Moreover, cancer prediction models are often not accurate enough to predict where the critical transitions will occur reliably, and there is usually a gap between theoretical results and clinical practice.

To address these issues, we have developed a novel but simple model that takes advantage of the distinctive differences in dissipation cost between cancer cells and normal cells. By analyzing the thermodynamic cost and the dynamical driving force, we can predict the critical transitions of cancer. We have used gastric cancer as a representative example, as it has one of the highest incidence rates among all cancers. Gastric cancer is a complex and heterogeneous disease that involves multiple genetic and epigenetic alterations. Models have been developed to study gastric cancer [2]. In 1965, Lauren divided gastric cancer into two types: the intestinal‐type and the diffuse‐type, which are separated by characteristic histological features [3]. Compared with the intestinal‐type, the diffuse‐type is more aggressive. Approximately 32% of the diffuse subtype tumor cells are poorly differentiated, indicating that the diffuse type of gastric cancer cells are more likely to be cancerous [4]. Typically, when diffuse gastropathy is diagnosed, it is already cancerous, as there are no well‐defined precursor lesions for diffuse gastric cancer (DGC) at present. Therefore, we have developed a model of DGC to identify early warning signals of cancer.

To investigate the role of genetic and epigenetic factors in the development of DGC, we conducted a literature research and constructed a gene regulatory network to analyze the molecular mechanisms involved. The network integrates information on both the genetic and epigenetic levels, allowing us to examine the interactions and influences of these factors on DGC. Using this network, we quantified the landscape of DGC and identified two stable states: a normal state and a cancer state. By examining the biological functions and gene expression levels associated with each state, we gained a system‐level understanding of the DGC formation and the development.

The use of entropy production rate (EPR) [5] and mean‐flux [6] as early warning signals for precancerous and pre‐metastasis formation in DGC offers a promising new approach. We found that these signals can detect critical transitions from the normal state to cancer state much earlier than traditional methods such as critical slowdown and flickering frequency. Furthermore, the use of gene expressions (simulated) over time to calculate the differences in cross‐correlations both forward and backward in time provides a feasible way to detect these drastic changes through the real‐time experiments or clinical trials for precancerous and premetastasis formations. The time‐irreversible measure can make it a useful tool for early diagnosis of cancer. Overall, these findings have significant implications for cancer research and clinical practice, as they provide a new way to detect cancer at an early stage when it is more treatable.

The findings from this study can be applied in clinical practice to detect early warning signals of DGC formation and development. By using gene expression data and calculating the entropy production rate and mean‐flux, we can detect the critical transitions from the normal state to the cancer state much earlier than other existing methods. This provides a feasible way to detect drastic changes in gene expression and could be used as an early warning system for clinicians to detect precancerous or premetastasis formations. Additionally, the gene regulatory network constructed in this study provides a more comprehensive understanding of the molecular mechanisms underlying DGC and can be used to guide future research and drug development. Overall, this study has the potential to contribute to the prevention and early detection of cancer, ultimately leading to improved patient outcomes.

## 2 MODELS AND RESULTS

### 2.1 Gene regulatory network and its dynamics for gastric cancer

**FIGURE 1:**
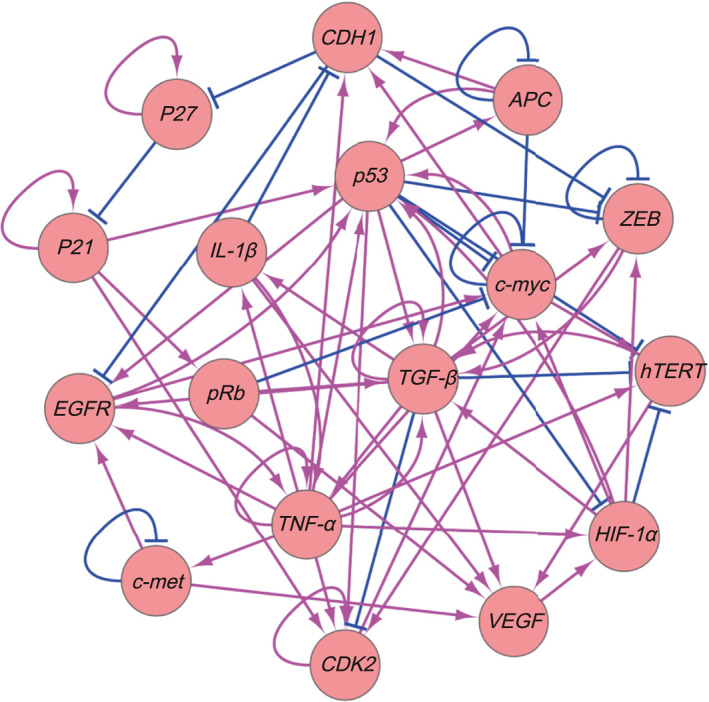
The gene regulatory network of the diffuse‐gastric cancer with 17 nodes and 70 regulations. (53 activations and 17 repressions. The arrows and the short bars represent the activating and the repressing regulations, respectively. The weights (regulation strengths) of the network and regulation type matrix can be found in Supporting Information Tables S4, S5).

In this study, it is interesting to note that the gene regulatory network for DGC includes both genetic and epigenetic influences, which can provide a more comprehensive understanding of the molecular mechanisms involved in DGC. In Figure 1, the arrows represent the active regulations and the short bars represent the repressive regulations. It is also important to consider the specific genes and characteristics of the different types of gastric cancer, such as the intestinal‐type and diffuse‐type. The inclusion of *CDH1* in the network is also relevant, given its known role in gastric cancer (the gene functions and network wiring can be found in Supporting Information Table S1, S2). While the other genes such as *k‐sam* may also be important for DGC, their interactions with the other 17 genes in the network have not been well‐established in the literature research, and therefore it might not be possible to include them in the analysis at this time. As the new research and methods become available, the gene regulatory network can be updated and expanded to include additional genes and interactions.

The dynamics of the gene regulatory network can be described by the ordinary differential equations (ODEs) [7, 8, 9, 10] as follows:

(1) $$\frac{dX_{i}}{dt}=F_{i}=g_{i}\prod_{j=1}^{n_{i}}H_{ji}-k_{i}X_{i}$$

Here, $\frac{dX_{i}}{dt}$ represents the gene expression or related protein concentration which changes with respect to time, parameter $g_{i}$ and $k_{i}$ represent the basal generation rate and the self‐degradation rate of the gene or protein, respectively. $X_{i}$ represents the gene expression level (the protein concentration) of the gene i. j is the subscript of the gene which regulates the gene *i*. *n*_*i*_ denotes the total number of the genes which regulate the gene i. $H_{ji}$ is a Hill function [11] defined as below:

(2) $$H_{ji}=\frac{{\left(S_{ji}\right)}^{n}}{{\left(S_{ji}\right)}^{n}+{\left(X_{j}\right)}^{n}}+{\left(\lambda_{ji}\right)}^{r}\frac{{\left(X_{j}\right)}^{n}}{{\left(S_{ji}\right)}^{n}+{\left(X_{j}\right)}^{n}}$$

In Equation (2), S is the “threshold” of the sigmoid function with the function of the half maximum value (parameter selection can be seen in the Supporting Information). The subscripts i and j are the same as those in Equation (1). n is the Hill coefficient and depicts the steepness of the sigmoid function which describes the synergy of the interactions. The parameter $\lambda_{ji}$ represents the regulation strength from $X_{j}$ to $X_{i}$ which must be greater than 1. The parameter *r* describes the regulation type. If r = +1 (r = −1), the regulation type is activation (inhibition).

In our model, the parameters are set as n = 4, $S_{ji}$ = 2.5, $g_{i}$ = 1 and $k_{i}$ = 1. The regulation weight $\lambda_{ji}$ of the network is a matrix which can be seen in Supporting Information Table S1. In Equation (1), *i* = 1, 2,… 17. Therefore, there are 17 ODEs used to describe the whole network. (The simulation value of gene expression can be seen in Supporting Information Table S3, parameter selection are in Supporting Information)

### 2.2 Potential landscape and kinetic paths of DGC

**FIGURE 2:**
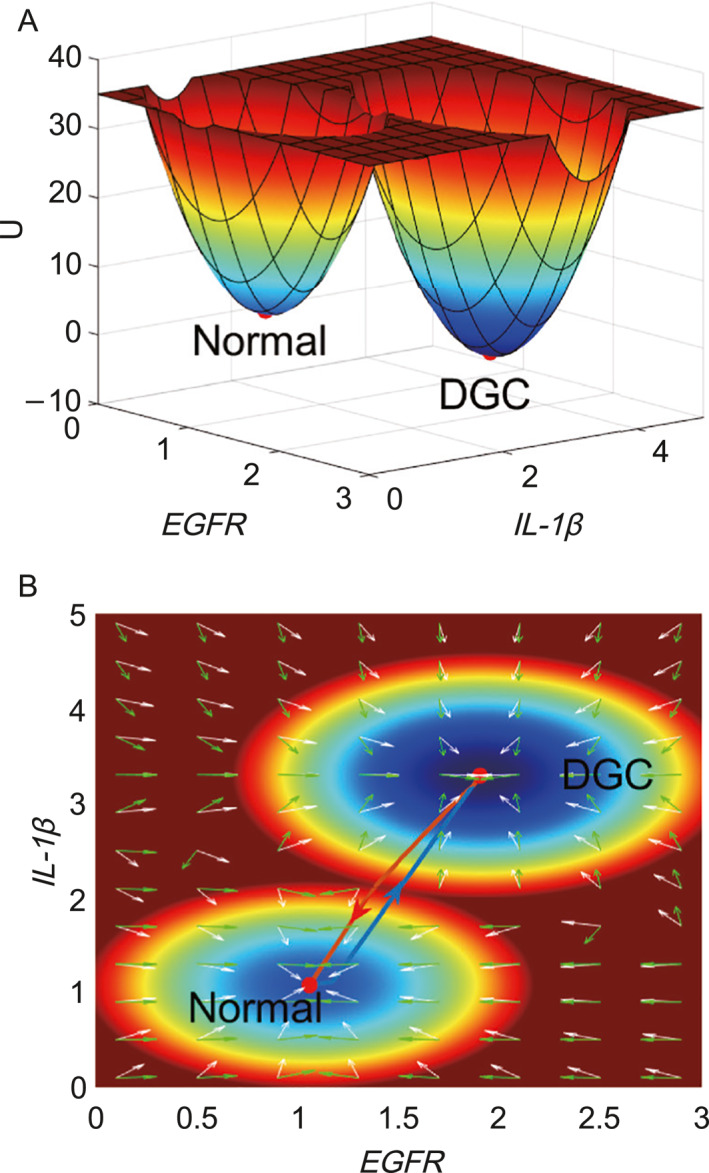
The two stable state landscape of the diffuse gastric cancer (DGC). (A) The three dimensional landscape and dominant kinetic paths. (B) The corresponding two dimensional landscape of the DGC. The lines in red, and blue represent the dominant kinetic path from the normal to the gastric cancer state and from the gastric cancer to the normal state. White arrows and green arrows represent the negative gradient of the potential landscape and the steady state probability curl flux force, respectively.

There are 17 genes in the gene regulatory network (Figure 1) of DGC. We can quantify 17‐dimensional probability distribution or landscape. The related potential landscape U was obtained according to the definition $U=-\ln\;P_{\text{ss}}$ [12, 13, 14]. $P_{\text{ss}}$ is the probability distribution of the steady‐states. To visualize the landscape clearly, we choose two relevant dimensions (*EGFR* and *IL‐1β*, these two genes have been extensively studied in DGC) by integrating out other dimensions. In Figure 2, *X*‐axis shows the expression level of *EGFR*, which is crucial in a variety of tumor development including DGC [15]. *EGFR* has close relationship with *CDH1*. Gene *CDH1* encodes the protein of E‐cadherin. Mutations of the *CDH1* extracellular domain may result in the alteration of its interaction with *EGFR* [16]. *Y*‐axis shows the expression level of *IL‐1β*. *IL‐1* cluster plays a significant role in immune response signaling pathways [17]. *IL‐1β* is related to the secretion of gastric acid, which can stimulate neoplastic growth [18].

The landscape of DGC has two stable state attractors, which correspond to the normal state and the cancer (DGC) state. These two states are defined based on the biological functions and gene expression levels of the 17 genes in the regulatory network. In the normal state, the tumor suppressor genes *P53*, *APC*, and *pRb* have high expression levels, while the gene expression level of *CDH1* is also high. In contrast, in the cancer state, *CDH1* is usually mutated, leading to a lower gene expression level. The other 14 (such as *VEGF* and *EGFR*) genes have high expression levels in the cancer state, while their expression levels are low in normal cells. The parameters in the model were determined by comparing the simulated results of gene expression levels in each state with experimental and clinical data. According to the results, the simulated gene expression levels are consistent with previous experimental literature, as shown in the Supporting Information Table S3.

In Figure 2, we can observe that the expression levels of *EGFR* and *IL‐1β* are low in the normal state, while they are high in the cancer state [19]. The barrier height from the normal to the cancer states is 28.7035, which is lower than the barrier height from the cancer to normal states (35.5352) at certain conditions (Figure S1. The landscape which contains three stable states project to genes *EGFR* and VEGF.). The barrier height is a measure of the difference in potential (reflecting the differences in probabilities) between the two stable state attractors, and it quantifies the degree of difficulty of switching between these states. The lower barrier height from the normal state to the cancer state implies that it is relatively easier to switch from the normal cell state to the cancer cell state. However, the higher barrier height from the cancer state to the normal state indicates that it is difficult to reverse back from the cancer state to the normal state. This result may explain why cancer is difficult to cure, as the attractor has to overcome the high barrier height in order to switchback to the normal state.

The path integral approaches [13, 20] were used to quantify the kinetic paths between the normal and cancer states. This approach involves calculating the probability of a transition between two states by summing over all possible paths that the system can take between those states. In this case, the paths represent the different combinations of gene expression levels that the system can take as it switches from the normal to the cancer state, or vice versa. By quantifying the dominant paths between the two states, we can gain insights into the underlying biological processes that drive the transition from one state to the other. The red path in Figure 2B represents the dominant kinetic path from the normal state to the cancer state, while the blue path represents the dominant kinetic path from the cancer state to the normal state. In a biological system, the driving force can be decomposed into a gradient force (represented by the white arrows) of the potential landscape and a rotational flux force (represented by the green arrows). The dominant paths are the result of the combined action of the forces from the gradient direction of the potential and the rotational curl flux. The dominant paths connecting the normal and cancer states are irreversible due to the rotational nature of the flux force [21]. This indicates that the processes of DGC formation and cancer reversion are independent and irreversible biological processes. In other words, it is difficult to reverse the cancer state back to normal because the dominant kinetic path from cancer to normal is separated from the dominant kinetic path from normal to cancer. The irreversible nature of the dominant paths also suggests that the underlying biological mechanisms of DGC formation and cancer reversion may differ, which can help to guide the development of targeted therapies for DGC.

### 2.3 Influences of the mutations of the key genes and the detection of the precancerous and metastasis early warning signals

Exploring mutations in key genes is crucial for observing changes in landscape topography and detecting critical thresholds approaching, as indicated by various bifurcations [22]. By studying these mutations, we can gain insights into the dynamic behavior of the system and anticipate potential transitions to critical states.

#### 2.3.1 CDH1

Diffuse‐type gastric cancer is characterized by poorly cohesive clusters of cells resulting from mutations of the *CDH1* gene, which encodes the E‐cadherin protein. Such mutations have been reported in more than 40 additional diffuse‐type gastric cancers [23]. Over two‐thirds of diffuse‐type gastric cancers have been suggested to be associated with identified *CDH1* germline mutations. Many studies with different populations have shown that 88% of patients diagnosed with DGC have a positive germline *CDH1* gene mutation. Moreover, 100% of *CDH1* mutation carriers have shown microscopic changes of the signet ring cell adenocarcinoma of the stomach [24].

In the context of gene regulatory networks, ODE modeling offers insights into how various factors influence system behavior. Here, the focus lies on exploring the impact of a *CDH1* mutation—a common occurrence in cancer that disrupts cell–cell adhesion regulation. The regulation force, denoted as $F\left(X_{i}\right)$ in the ODEs, embodies the collective effect of elements governing *CDH1* gene expression. By adjusting the regulation force value E (${F_{i}}^{\prime }=F_{i}*E$, where i = 17 denotes the *CDH1* gene), the model simulates the effects of a *CDH1* mutation on regulatory strength.

The accompanying figure depicts shifts in landscape topography and bifurcation behavior as the regulation strength E varies. As E diminishes, the system transitions from a monostable state (representing normal cellular behavior) to a bistable state, where the emergence of a cancer state becomes feasible. Ultimately, with decreasing regulation strength, the cancer state predominates, highlighting the profound impact of *CDH1* mutations on cellular dynamics.

**FIGURE 3:**
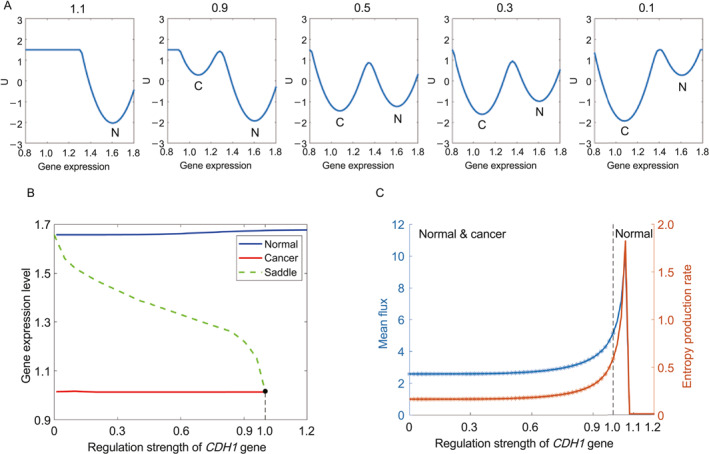
The simulation of the diffuse gastric cancer (DGC) upon the mutations of the *CDH1* gene. (A) The variations of the landscape topography in 2 dimensions. The label N and C represent the normal and cancer state, respectively. (B) The bifurcation of the landscapes when the regulation strengths change. The lines in color blue and red represent the steady state points in the normal state and the cancer state when regulation strength changed. The dotted line in color green represents the fixed point at the saddle when regulation strength changed. (C) The Entropy Production Rate (the orange line) and Mean flux (the blue line) change when the regulatory force of regulation strength (*CDH1*) value changed from 0 to 1.5. The red and blue stars represent normal and cancer state, respectively.

In Figure 3B, the bifurcation behavior of the system is depicted as the regulation strength E varies. Blue and red lines represent the steady state attractors of the normal and cancer states, respectively, while a dotted green line denotes unstable states. Initially, when the value of E exceeds 1, the system exhibits only one stable state, representing the normal state (as observed at the regulation strength value of 1.1 in Figure 3A). However, upon reducing the regulation strength E to 1.0, a bifurcation occurs, giving rise to a second stable state—representing the cancer state (as evident at the regulation strength value of 0.9 in Figure 3A). Subsequently, as the value of E continues to decrease, the system transitions to a regime characterized by a single stable state, namely, the cancer state (as demonstrated at the regulation strength value of 0.1 in Figure 3A). This underscores the pivotal role of regulation strength E indictating the system’s behavior and its propensity for transitioning to a cancerous state.

In Figure 3C, additional insights into the system behavior are presented, showcasing the mean‐flux and entropy production rate (EPR) values across different regulation strength E settings. When E ranges from 1.1 to 1.5, reflecting the regime where only the normal state prevails, both EPR and mean‐flux values remain low. However, as the regulation strength E decreases toward 1.0, indicative of the emergence of the cancer state, a notable bifurcation occurs. At this juncture, a sharp increase in both mean‐flux and EPR is observed, occurring earlier than the actual bifurcation points. This surge signifies a significant escalation in the nonequilibrium driving force and the thermodynamic cost essential for maintaining the system’s stability. Consequently, monitoring these signals becomes pivotal for detecting cancer onset at its nascent stage, thereby facilitating timely intervention and treatment strategies.

In the DGC system, the presence of nonzero curl flux disrupts detailed balance, instigating a force that propels the system out of equilibrium. While the conversive nature of the gradient force aids in maintaining state stability, the curl flux tends to circumnavigate around the attractor point rather than converging directly toward it. Consequently, this flux tendency destabilizes the point attractor [25]. The entropy production rate (EPR) serves as a metric to gauge the thermodynamic cost or dissipation required to uphold the system’s steady state over time. A sharp increase in EPR indicates a substantial surge in thermodynamic consumption, particularly in the presence of the cancer state. Furthermore, the dissipation characteristics of cancer cells markedly differ from those of normal cells. Cancer cells often undergo metabolic reprogramming, shifting from aerobic respiration to glycolysis [11, 26]. This metabolic alteration results in elevated energy consumption by cancer cells, facilitating functions such as angiogenesis, proliferation sustenance, apoptosis resistance, and other vital processes [27].

The EPR and mean‐flux serve as valuable indicators for detecting bifurcations and critical transitions in the DGC system, particularly when the system approaches a bifurcation point or when a new state, such as the cancer state, emerges. A significant increase in EPR and mean‐flux values signifies the onset of a new state, offering crucial insights into the system’s behavior, metabolic alterations, and the associated thermodynamic costs. These indicators play a pivotal role in understanding the dynamics of the DGC system and can aid in timely intervention and treatment strategies.

#### 2.3.2 TGF‐β

Research on TGF‐β has garnered increasing attention due to its involvement in various facets of cancer, including cancer cell proliferation, epithelial‐mesenchymal transition, angiogenesis, and invasion of gastric cancer [28, 29]. TGF‐β and TGF‐β receptor antagonists are widely employed in clinical trials for the treatment of solid tumors, including diffuse‐type gastric cancer (DGC) [30]. Excessive TGF‐β expression can exacerbate DGC progression [31]. To investigate the impact of alterations in TGF‐β expression on DGC progression, we simulated TGF‐β overexpression or loss using a similar method. In modeling the dynamics of the gene regulatory network with ODEs, we modified the regulatory force $F\left(X_{i}\right)$ for gene *i* = 6 (representing the *TGF‐β* gene) to ${F_{i}}^{\prime }=F_{i}*C$, mimicking the effects of mutations. We varied the regulation strength C from 1.5 to 0 to simulate the transition from large to small regulation strength until it vanished (mutation).

**FIGURE 4:**
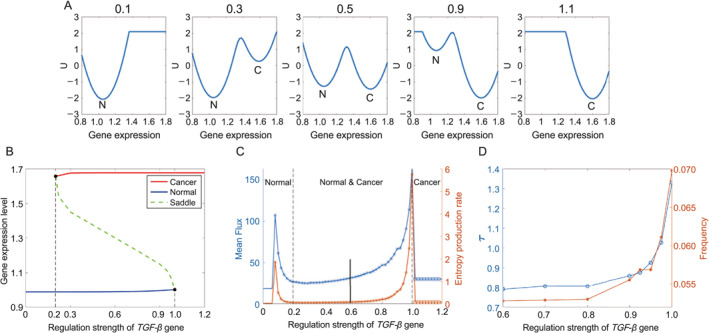
The simulation of the diffuse gastric cancer (DGC) upon the mutations of the *TGF‐β* gene. (A) The variations of the landscape topography in 2 dimensions. The label N and C represent the normal and cancer state, respectively. (B) The bifurcation of the landscapes when the regulation strengths change. The lines in color blue and red represent the steady state points in the normal state and the cancer state when regulation strength changed. The dotted line in color green represents the fixed point at the saddle when regulation strength changed. (C) The Entropy Production Rate (the orange line) and Mean flux (the blue line) change when the regulatory force of regulation strength (*TGF‐β*) value changed from 0 to 1.5. The red star and blue circle represent normal and cancer state, respectively. (D) The critical slowdown and flickering frequency variations by changing the regulatory force of regulation strength of *TGF‐β*.

Figure 4 illustrates the changes in landscape topography and bifurcation as the regulation strength varies. As depicted in Figure 4A, with increasing regulation strength C, the landscape transitions from a monostable state (normal) to a bistable state (normal and cancer), eventually settling into a monostable state (cancer).

Figure 4B illustrates the landscape bifurcation as the regulation strength C varies. The blue and red lines represent the steady‐state attractors for the normal and cancer states, respectively, while the green dotted line signifies the unstable intermediate state resulting from changes in regulation strength. Initially, with C below 0.2, only one normal state is observed (as depicted at C = 0.1 in Figure 4A). As C surpasses 0.2, a bifurcation occurs, marking the emergence of the cancer state (as indicated at C = 0.3 in Figure 4A). Subsequently, with further increases in C, the prominence of the cancer state intensifies (as shown at C = 0.9 in Figure 4A). Approaching C = 1, another bifurcation arises, leading to the disappearance of the normal state and leaving only the cancer state (as seen at C = 1.1 in Figure 4A).

In Figure 4C, it’s evident that when the regulation strength C is below 0.1 (indicating only one normal state), the mean‐flux and EPR values remain low and stable. As the regulation strength C approaches 0.2, both mean‐flux and EPR start to rise, reaching their initial peak at approximately 0.1. This rise correlates with the bifurcation from a system characterized by a single normal state to one with the coexistence of normal and cancer states. Such a transition entails significant changes in dynamical driving force and thermodynamic cost, which are effectively captured by mean‐flux and EPR. These fluctuations act as early warning signals for precancerous states. As the regulation strength C is greater than 0.2, mean‐flux and EPR exhibit a gradual decline but undergo a sharp surge near the second bifurcation point (at C = 1.0). At this stage, the normal state vanishes, leaving only the cancer state. This drastic change in system consumption and nonequilibrium signifies a critical phase where mean‐flux and EPR peaks can indicate pre‐metastatic conditions, suggesting that cancer has progressed toward a highly challenging‐to‐treat and potentially fatal direction.

The DGC system is regulated by a complex network that can rapidly switch to a contrasting state near a critical point under the influence of small stochastic perturbations. Tipping point detection methods, such as critical slowdown, have been applied across various fields, including climatic systems, financial markets, ecosystems, and systems biology [32, 33]. Additionally, flickering frequency has been used to detect early warning signals in previous studies [22, 34]. The flickering frequency method is derived from the reciprocal of the mean first passage time [35]. Critical slowdown and flickering frequency characterize different aspects of tipping points, namely, relaxation or response time and transition rate, respectively.

From Figure 4D, it is clear that critical slowdown and flickering frequency begin to increase at a value of 0.8 and reach their peak at 1. In this range, the normal state becomes increasingly flat and unstable, while the cancer state becomes more dominant. Critical slowdown and flickering frequency indicate that the normal state is on the verge of disappearing, with the basin of attraction becoming very flat. In contrast, variations in EPR and mean flux can be observed much earlier, with mean flux starting to increase at approximately 0.5 and EPR at around 0.6. These earlier indicators are crucial in clinical settings, as they enable the initiation of preventive and treatment measures at the earliest possible stage.

Compared to critical slowdown and flickering frequency, the mean flux and EPR methods can detect bifurcation points significantly earlier during phase transitions. While critical slowdown can become pronounced near the critical point, causing the steady‐state to flatten and the system’s recovery ability to diminish, this period is brief and challenging for effective intervention. Therefore, identifying early warning signals and implementing timely interventions is crucial to prevent catastrophic diseases. The dynamic driving force, quantified by mean flux, and the thermodynamic cost, measured by EPR, can provide early indicators far ahead of traditional predictive methods.

Taking the gene *TGF‐β* in the DGC system as an example, both mean flux and EPR show their first peaks well before the bifurcation point where the cancer state emerges. This early warning can signal the precancerous stage, allowing for preventive measures against cancer development. Similarly, the second peaks of mean flux and EPR occur well before the bifurcation point where the normal state disappears and the cancer state becomes dominant. Once the normal state vanishes, the DGC system’s likelihood of recovery diminishes significantly. These early warning signals are crucial for preventing cancer metastasis, emphasizing the importance of proactive measures in cancer management.

In summary, the mean flux and EPR can quantify the nonequilibrium flux force and thermodynamic consumption in the DGC system, particularly during the transition from a single normal state to a coexistence of normal and cancerous states. Both mean flux and EPR exhibit peaks well before the bifurcation point, providing quantitative early warning signals for critical phase transitions toward disease progression. These early warning signals can aid in preventing catastrophic diseases and improving healthcare outcomes.

### 2.4 Quantifications of the time irreversibility and early warning signal from the stochastic gene expression time traces

Although the mean flux and EPR can quantify early warning signals for the DGC system, measuring these values in cancer systems can be challenging. Energy is required to sustain the cell’s steady state in cancer systems, which breaks detailed balance and time reversal symmetry. To bridge the gap between theory and clinical practice, we developed a new approach to quantify nonequilibrium dynamics in cancer systems. With the rapid development of advanced technology, time series data of gene expressions during cancer development will become available. This data can be used to extract information on time asymmetry directly from experimental time series. Consequently, this information can be utilized to characterize nonequilibrium and provide early warning signals for cancer progression.

We calculated the differences between forward and backward two‐gene cross‐correlation functions of simulated stochastic gene expression trajectories over time to quantify time irreversibility and thus the degree of nonequilibrium. These stochastic gene expression trajectories can be obtained from simulations consistent with experimental data trends. This method allows us to quantify the nonequilibrium dynamics in cancer systems without directly measuring the mean flux or EPR, which may be difficult to detect in practice.

The cross‐correlation between two genes X and Y can be defined as $C_{\text{XY}}\left(\tau \right)=\langle X\left(0\right)Y\left(\tau \right)\rangle$ = $\sum X_{i}Y_{j}P_{i}^{ss}P_{ij}$, where τ represents the time interval. If the gene X and Y are specific to the states A and B, we can quantify time irreversibility by calculating the difference between forward and backward cross‐correlation functions in time. This difference is expressed as ${\;C}_{\text{XY}}\left(\tau \right)$ − $C_{\text{YX}}\left(\tau \right)=X^{A}Y^{B}\left[P_{A}^{ss}P_{\text{AB}}-P_{B}^{ss}P_{\text{BA}}\right]$, which is related to the flux from state A to state B [36, 37]. During a brief time span, $\tau P_{ij}\left(\tau \right)\approx k_{ij}\tau$ and $P_{i}^{ss}k_{ij}-P_{j}^{ss}k_{ji}=J_{ij}^{ss}\neq 0$. $J_{ij}$ represents precisely the nonequilibrium steady‐state probability flux from state i to j. Hence, the distinction between the forward and backward cross‐correlation functions is directly linked to the flux $J_{ij}^{ss}$. Hence, ${\;C}_{\text{XY}}\left(\tau \right)$ − $C_{\text{YX}}\left(\tau \right)=X^{A}Y^{B}J_{\text{AB}}^{ss}\tau$ and $J_{AB}^{ss}$ = $\frac{1}{X^{A}Y^{B}}\lim_{\tau \to 0}\frac{{\;C}_{\text{XY}}\left(\tau \right)-C_{\text{YX}}\left(\tau \right)}{\tau }$. $J_{\text{AB}}^{ss}$ represents the flux from state A to state B, which is related to mean‐flux. Both EPR and mean‐flux can quantify their reversibility and degree of nonequilibrium.

**FIGURE 5:**
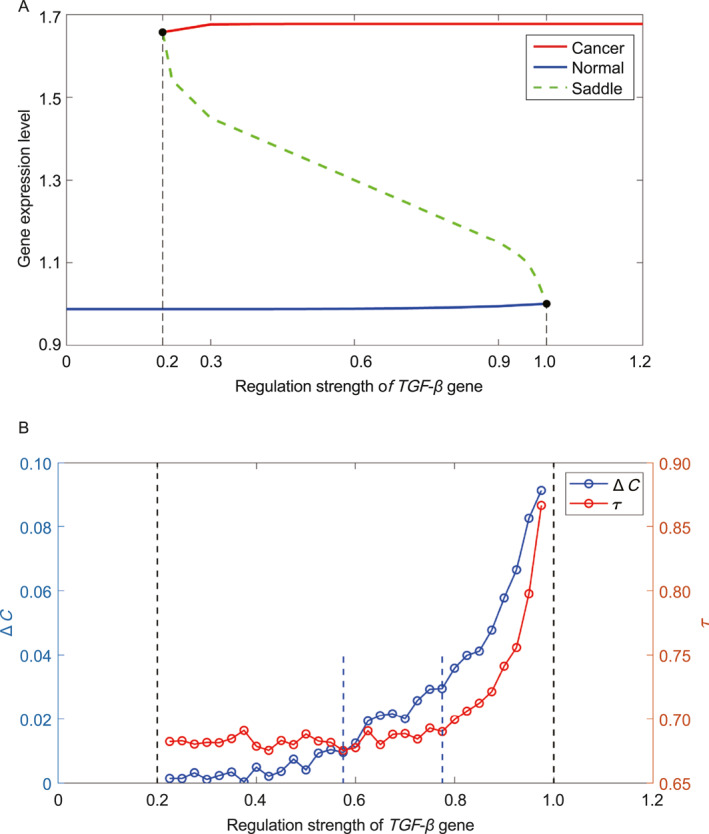
The simulation of the difference of the forward and backward cross correlation functions Δ*C* and *τ*. (A) The bifurcation of the landscapes when the regulation strengths change. (B) The difference of the forward and backward cross correlation functions Δ*C* and *τ* when changing the regulation strengths of gene *TGF‐β*. The blue and red lines represent the Δ*C* and *τ* variations when changing the regulation strength of gene *TGF‐β*.

In Figure 5B, we present the differences in cross correlations Δ*C* as the regulation strength changes. The regulation strength values used here are the same as those in Figure 4C. The quantity Δ*C* is defined as $∆C=\sqrt{\frac{1}{t_{f}}\int_{0}^{t_{f}}{\left(C_{\text{XY}}\left(\tau \right)-C_{\text{YX}}\left(\tau \right)\right)}^{2}d\tau }$, which quantifies the difference in cross correlations between forward and backward time. In Figure 5B, the blue and red lines represent the changes in Δ*C* and critical slowdown, respectively, as the regulation strength varies. The Δ*C* begins to increase at a regulation strength of approximately 0.5775, while the critical slowdown starts to rise at about 0.7775, indicating that the Δ*C* change occurs significantly earlier. It is also evident that the Δ*C* values are positively correlated with mean flux and the EPR when the regulation strength of *TGF‐β* changes [37]. Since gene expression trajectories are stochastic in nature, there are fluctuations in the Δ*C* values. We can use Δ*C* to characterize changes in the mean flux and the EPR.

Our DGC model can be used to quantitatively analyze cancer systems and identify early warning signals of cancer through the cross‐correlation of gene expression values, thereby better integrating clinical experiments with theoretical calculations. By analyzing the trajectories of simulated or experimentally obtained real‐time gene expressions, it is possible to calculate the difference in cross‐correlations in forward and backward directions over time. The time irreversibility, as reflected by the sharp variations in the cross‐correlation function, can serve as an early warning signal of DGC in clinical or experimental settings. Additionally, the initial increase in cross‐correlations Δ*C* for the two regulation strengths occurs at a value no later than 0.8, providing an early detection opportunity for DGC prevention or treatment.

In summary, our approach quantifies time irreversibility in cancer systems by calculating the differences between forward and backward cross‐correlation functions directly from real‐time traces, without directly measuring the EPR or mean flux. If real‐time series data and network wiring information are available, we can also obtain EPR and mean flux to quantify the irreversibility and degree of nonequilibrium [38]. Our method provides a practical way to identify early warning signals for critical phase transitions in cancer systems. Therefore, this new approach offers a valuable tool for studying nonequilibrium dynamics in cancer systems and aids in the identification of early warning signals for critical transitions toward cancer.

## 3 CONCLUSIONS

Detecting early warning signals for cancer is crucial for timely diagnosis and treatment. Although current cancer prediction models are not always precise, our study introduces a novel and straightforward method for early cancer diagnosis by identifying early warning signals. We focused on diffuse‐type gastric cancer (DGC) as a case study. By calculating real‐time traces and thermodynamic costs (in terms of EPR), we identified precancerous or pre‐metastasis points. We compared our findings with other established prediction methods, such as critical slowdown and flickering frequency, within the DGC system. While these traditional methods can also predict the emergence of bifurcation points, our results based on mean flux and EPR provide warning signals significantly earlier. Hence, quantifying nonequilibrium changes using mean flux and EPR can serve as early warning indicators for the DGC system.

To bridge the gap between theoretical results and clinical practice, we calculated the time irreversibility of cross correlations for gene expression time series. Interestingly, our results showed a positive correlation with mean flux and EPR values. This correlation offers a practical approach and a new perspective for developing clinical diagnostic methods by detecting early warning signals directly from observational time series.

## 4 METHODS

### 4.1 Decomposition of the driving force into a landscape gradient and curl flux

A gene regulatory network can consist of the genes and the gene regulations. In our system, we use a *n* component vector *x* = (*x*_1_ *, x*_2_, …, *x*_*n*_) to represent the genes in the network. Here, *n* represents the gene amount and *x*_*i*_ (*i* = 1, 2, …*, n*) can be used to represent the related gene expressions (or protein concentrations) in the model. To describe the deterministic dynamics of the network, there will be *n* ODEs ($\dot{x}$ = F(*x*)). Where F(*x*) denotes the deterministic driving force.

In the biological system, the intrinsic and external fluctuations both exist. We added a term *ξ*(x*, t*) of the ODEs ($\dot{x}$ = F(*x*)) to represent the stochastic dynamics of the system as: $\dot{x}$ = F(*x*) + *ξ*(x*, t*). These stochastic differential equations are called Langevin equations. The term of *ξ*(x*, t*) represents the fluctuations and stochastic force and the related correlation function is quantified as 〈*ξ*(x*, t*)〉 = 0 and 〈*ξ*(x*, t*)*ξ* ^*T*^(x*, t* ^′^)〉 = 2D(*x*)*δ*(*t* − *t* ^′^). D(*x*) is the diffusion coefficient matrix which characterizes the correlation and fluctuation strength.

Based on the Langevin dynamics simulation, we can collect the statistics from the simulated trajectories and obtained the corresponding probability (*P*(*x*, *t*)) by the Fokker–Planck (diffusion) equation: *∂P/∂t* = − ∇ · [F*P*− ∇ · (D*P*)]. It can be rewritten as: *∂P/∂t* = − ∇ · *J*, where *J* is used to denotes the probability flux. In a steady state, *∂P*_*ss*_ */∂t* = ∇ · *J*_*ss*_ = 0. The probability flux of the steady state can be given as *J*_*ss*_ = F*P*_*ss*_ −D · ∇ *P*_*ss*_. In a nonequilibrium system net flux is nonzero; therefore, the detailed balance is explicitly broken or nonequilibrium can be measured by the net flux (∇ · *J*_*ss*_ ≠ 0). The driving force *F* can be then decomposed into [39]: *F* = −D · ∇ *U* + *J*_*ss*_ */P*_*ss*_. Where −D · ∇ *U* quantifies the gradient driving force of the potential landscape and *J*_*ss*_ */P*_*ss*_ quantifies the steady state probability flux force of the steady state. The potential landscape can be obtained by *U* = −ln*P*_*ss*_.

### 4.2 Self‐consistent mean field approach

The self‐consistent mean field approach [40] can be utilized to solve the Fokker–Planck equation in high‐dimensional space. If the probability distribution is converted to individual probabilities as *P*(*x*_1_ *, x*_2_, ···*, x*_*n*_ *,t*) ∼ _∏*i*_ *P*(*x*_*i*_ *, t*), the functions can be solved self‐consistently. This reduction in system dimensionality from *m* ^*n*^ to *m* × *n*, significantly enhances computational feasibility, where *m* represents the number of possible values for variable *x*.

Additionally, the Gaussian Probability Distribution Ansatz can provide a useful approximation. For small diffusion coefficients *D*, the equations are given by the following equations:

(3) $$\dot{\bar{x}}\;\left(t\right)=F\left(\bar{x}\left(t\right)\right)$$

(4) $$\dot{\sigma }\left(t\right)=A\left(t\right)\sigma \left(t\right)+\sigma \left(t\right)A^{T}\left(t\right)+2D\left(\bar{x}\left(t\right)\right)$$

Here, $\bar{x}$(*t*) and σ(*t*) denote the mean vector and the covariance matrix, respectively. The matrix A has elements represented as $A_{ij}\left(t\right)=\frac{\partial F_{i}\left(\bar{x}\left(t\right)\right)}{\partial {\bar{x}}_{j}\left(t\right)}$ . In this study, we consider only the diagonal elements of *σ*(*t*). Following the Gaussian Approximation, the probability distribution evolution of each *x*_*i*_ can be described as follows:

(5) $$P\left(x_{i},t\right)=\frac{1}{\sqrt{2\pi \sigma_{i}\left(t\right)}}\exp\left\{-\frac{{\left[x_{i}-\bar{x_{i}}\left(t\right)\right]}^{2}}{2\sigma_{i}\left(t\right)}\right\}$$

For a monostable system, the probability distribution can be obtained based on Equation (5). In a multistable system, with multiple fixed points, the total distribution is a combination of Gaussian distributions. The probability distribution for a multi‐steady state system is: *P*(*x,t*) = ∑*ω*_*i*_ *P*_*i*_(*x*). Where *ω*_*i*_ represents the weight of each separate state. Therefore, the final probability distribution for a multi‐steady state system within the landscape is described as: *U*(*x*) = −ln*P*_*ss*_(*x*).

### 4.3 The path integral approach

We derived the dynamics of the transition probability based on the Onsager–Machlup functional [13], which can be quantified using the path integral approach: *P*(*x*_fin_ *,t*_f_; x_ini_ *,t*_i_) = ∫D[x(t)]exp{−S[x(t)]} = ∫D[x(t)]exp{−∫L(x(t))dt}. Here *x*_ini_ represents the initial state at time *t*_i_ and *x*_fin_ represents the final state at time *t*_f_. The Lagrangian is described as: $L\left(x\left(t\right)\right)=\frac{1}{4}\left(\dot{x}-F\left(x\right)\right)\cdot D^{-1}\cdot$ ($\dot{x}-F\left(x\right)$)+ $\frac{1}{2}$ ∇⋅F(x). The probability weight is determined by the action *S*[*x*(*t*)] = ∫L(x(t))dt. The notation ∫D[x(t)] depicts all possible paths from the initial state to the final state. Since each path is exponentially weighted, the dominant paths are identified as those with the maximum probability, or the least action *S*[*x*(*t*)]. In nonequilibrium systems, the presence of *J*_*ss*_ cannot be ignored. Therefore, the dominant kinetic paths will be distinct and irreversible in a nonequilibrium system.

### 4.4 Mean‐flux and entropy production

In a system operating away from equilibrium, a crucial inquiry revolves around gauging the extent of deviation from equilibrium. This can be delineated by various specific metrics. As previously discussed, the presence of a nonzero, yet divergence‐free rotational flux serves as a distinctive marker of nonequilibrium systems, closely linked to the existence of nonequilibrium energy pumps. This flux plays a pivotal role in sustaining the stability of limit cycle oscillations within such systems [36, 37, 39, 41]. Inspired by this premise, we can introduce the concept of an average probabilistic flux $J_{\text{average}}=\frac{\int \left|J_{ss}\right|dx}{\int dx}$ to quantify the overall nonequilibrium magnitude of a system. Furthermore, energy dissipation and consumption are inherent features of nonequilibrium systems. The energy dissipation, directly related to the total entropy production rate of the system and environment, serves as a global physical metric to gauge the system’s deviation from equilibrium. For nonequilibrium systems, the temporal change in system entropy can be dissected into two components: $\frac{dS}{dt}=\frac{d}{dt}\int P\left(x,t\right)\ln\;P\left(x,t\right)$, $dx=\int \frac{\left(J\cdot {\left(DD\right)}^{-1}\cdot J\right)}{P}dx-\int \left(J\cdot {\left(DD\right)}^{-1}\cdot F_{\text{eff}}\right)dx=S_{t}^{\prime }-S_{e}^{\prime }$, where the entropy production rate (EPR), $S_{t}^{\prime }=$ $\int \frac{\left(J\cdot {\left(DD\right)}^{-1}\cdot J\right)}{P}dx$ is always non‐negative, representing the overall entropy change of the system and its surroundings, adhering to the second law of thermodynamics. Conversely, the heat dissipation rate or entropy flow rate $S_{e}^{\prime }$ = $\int (J\cdot {\left(DD\right)}^{-1}\cdot \left(F-D\nabla \cdot D\right)dx$ can either be positive or negative, accounting for energy and information exchange between the system and its environment, leading to a rise or fall in the system’s entropy. Consequently, the entropy of a nonequilibrium system does not necessarily exhibit an increasing or maximized trend, while the total entropy production remains consistently positive. The effective force $F_{\text{eff}}$ is defined as $F_{\text{eff}}=F-D\nabla \cdot D$.

## AUTHOR CONTRIBUTIONS

**Chong Yu**: Conceptualization; data curation; formal analysis; funding acquisition; methodology; validation; writing ‐ original draft; writing ‐ review & editing. **Wenbo Li**: Data curation. **Xiaona Fang**: Conceptualization; supervision. **Jin Wang**: Conceptualization; project administration; supervision; writing ‐ review & editing.

## ACKNOWLEDGEMENTS

This study was supported by the National Natural Science Foundation of China (Grant Nos. 12205114 and 12234019) and the Natural Science Foundation of Jilin Province, China (Grant No. YDZJ202401385ZYTS).

## CONFLICT OF INTEREST STATEMENT

The authors declare that no competing interests exist.

## ETHICS STATEMENT

The authors declare that the study does not include animal and human experiments that violate ethics.

## Supporting information

Supplementary Material

## Data Availability

The data that support the findings of this study are available from the corresponding author upon reasonable request.
