# Supplementary material for: Identifying early warning signals of cancer formation
Source: Quant Biol. 2025 Jan 23;13(2):e81. doi: 10.1002/qub2.81 (PMC12806070; doi:10.1002/qub2.81)
Supplement: Supplementary file 1 — Supplementary Material [file QUB2-13-e81-s001.docx]

# Identifying early warning signals of cancer formation

Chong Yu,^1^*^,^*^2^ Wenbo Li^1^ , Xiaona Fang ^3∗^, Jin Wang^4∗^

1.State Key Laboratory of Electroanalytical Chemistry, Changchun Institute of Applied Chemistry, Chinese Academy of Sciences, Changchun, Jilin, China

# 2.Department of Statistics,JiLin University of Finance and Economics, Changchun, Jilin, China

3. Department of Chemistry, Northeast Normal University, Changchun, Jilin, China

# 4.Department of Chemistry and of Physics and Astronomy, State University of New York at Stony Brook, New York, USA

*Corresponding Authors: [fangxn482@nenu.edu.cn](mailto:fangxn482@nenu.edu.cn);jin.wang.1@stonybrook.edu

# Supplemental Items

# S1 Table: literature search results. These literature references are mainly from EVEX database. *a* represents activation and *r* represents repression. The parameters are the network parameters of the model we constructed.

|  | **Source gene** | **Target gene** | **Regulation type** | **literature** |
| --- | --- | --- | --- | --- |
| 1 | p53 | c-myc | r | [[1](#_ENREF_1)] |
| 2 | p53 | HIF-1α | r | [[2](#_ENREF_2)] |
| 3 | p53 | ZEB | r | [[3](#_ENREF_3)] |
| 4 | p53 | TGF-β | a | [[4](#_ENREF_4)] |
| 5 | p53 | APC | a | [[5](#_ENREF_5)] |
| 6 | p53 | EGFR | a | [[6](#_ENREF_6)] |
| 7 | p53 | hTERT | r | [[7](#_ENREF_7)] |
| 9 | p53 | CDK2 | a | [[8](#_ENREF_8)] |
| 10 | c-myc | p53 | a | [[9](#_ENREF_9)] |
| 11 | c-myc | c-myc | r | [[10](#_ENREF_10)] |
| 12 | c-myc | Bcl-2 | r | [[11](#_ENREF_11)] |
| 13 | c-myc | hTERT | a | [[12](#_ENREF_12)] |
| 14 | c-met | EGFR | a | [[13](#_ENREF_13)] |
| 15 | c-met | VEGF | a | [[14](#_ENREF_14)] |
| 16 | c-met | c-met | r | [[15](#_ENREF_15)] |
| 17 | HIF-1α | p53 | a | [[16](#_ENREF_16)] |
| 18 | HIF-1α | c-myc | a | [[17](#_ENREF_17)] |
| 19 | HIF-1α | ZEB | a | [[18](#_ENREF_18)] |
| 20 | HIF-1α | TGF-β | a | [[17](#_ENREF_17)] |
| 21 | HIF-1α | hTERT | r | [[19](#_ENREF_19)] |
| 22 | Bcl-2 | CDK2 | a | [[8](#_ENREF_8)] |
| 23 | Bcl-2 | HIF-1α | a | [[20](#_ENREF_20)] |
| 24 | Bcl-2 | Bcl-2 | a | [[21](#_ENREF_21)] |
| 25 | Bcl-2 | p53 | r | [[22](#_ENREF_22)] |
| 26 | Bcl-2 | Bcl-2 | a | [[21](#_ENREF_21)] |
| 27 | Bcl-2 | CDK2 | a | [[8](#_ENREF_8)] |
| 28 | APC | c-myc | r | [[23](#_ENREF_23)] |
| 29 | APC | APC | r | [[24](#_ENREF_24)] |
| 30 | APC | p53 | a | [[25](#_ENREF_25)] |
| 31 | APC | E-cadherin | a | [[26](#_ENREF_26)] |
| 32 | TGF-β | p53 | a | [[27](#_ENREF_27)] |
| 33 | TGF-β | IL-1β | a | [[28](#_ENREF_28)]. |
| 34 | TGF-β | TNF-α | a | [[29](#_ENREF_29)] |
| 35 | TGF-β | TGF-β | a | [[30](#_ENREF_30)] |
| 36 | TGF-β | ZEB | a | [[31](#_ENREF_31)] |
| 37 | TGF-β | VEGF | a | [[32](#_ENREF_32)] |
| 38 | TGF-β | hTERT | r | [[33](#_ENREF_33)] |
| 39 | TGF-β | EGFR | a | [[34](#_ENREF_34)] |
| 40 | TGF-β | CDK2 | r | [[35](#_ENREF_35)] |
| 41 | TNF-α | IL-1β | a | [[36](#_ENREF_36)] |
| 42 | TNF-α | Bcl-2 | a | [[37](#_ENREF_37)] |
| 43 | TNF-α | TNF-α | a | [[38](#_ENREF_38)] |
| 44 | TNF-α | HIF-1α | a | [[39](#_ENREF_39)] |
| 45 | TNF-α | c-met | a | [[40](#_ENREF_40)]. |
| 46 | TNF-α | P53 | a | [[41](#_ENREF_41)] |
| 47 | TNF-α | TGF-β | a | [[42](#_ENREF_42)]. |
| 48 | TNF-α | c-myc | a | [[43](#_ENREF_43)] |
| 49 | TNF-α | EGFR | a | [[44](#_ENREF_44)] |
| 50 | TNF-α | hTERT | a | [[45](#_ENREF_45)]. |
| 51 | TNF-α | CDK2 | a | [[46](#_ENREF_46)] |
| 52 | ZEB | ZEB | r | [[47](#_ENREF_47)] |
| 53 | ZEB | TGF-β | a | [[48](#_ENREF_48)] |
| 54 | ZEB | CDK2 | a | [[49](#_ENREF_49)]. |
| 55 | EGFR | TNF-α | a | [[50](#_ENREF_50)] |
| 56 | EGFR | p53 | a | [[51](#_ENREF_51)] |
| 57 | EGFR | c-myc | a | [[52](#_ENREF_52)] |
| 58 | VEGF | HIF-1α | a | [[53](#_ENREF_53)] |
| 59 | hTERT | VEGF | a | [[54](#_ENREF_54)]. |
| 60 | hTERT | TGF-β | a | [[55](#_ENREF_55)] |
| 61 | CDK2 | c-myc | a | [[56](#_ENREF_56)] |
| 62 | CDK2 | CDK2 | a | [[57](#_ENREF_57)] |
| 63 | IL-1β | E-cadherin | r | [[58](#_ENREF_58)] |
| 64 | IL-1β | VEGF | a | [[59](#_ENREF_59)]. |
| 65 | IL-1β | TNF-α | a | [[60](#_ENREF_60)] |
| 66 | P21 | CDK2 | a | [[57](#_ENREF_57)] |
| 67 | P21 | p53 | a | [[61](#_ENREF_61)] |
| 68 | P21 | pRb | a | [[62](#_ENREF_62)] |
| 69 | P21 | P21 | a | [[63](#_ENREF_63)] |
| 70 | P27 | P27 | a | [[64](#_ENREF_64)] |
| 71 | P27 | P21 | r | [[65](#_ENREF_65)] |
| 72 | pRb | TGF-β | a | [[66](#_ENREF_66)] |
| 73 | pRb | VEGF | a | [[67](#_ENREF_67)] |
| 74 | pRb | c-myc | r | [[68](#_ENREF_68)] |
| 75 | E-cadherin | ZEB | r | [[69](#_ENREF_69)] |
| 76 | E-cadherin | EGFR | r | [[70](#_ENREF_70)] |
| 77 | E-cadherin | P27 | r | [[71](#_ENREF_71)] |
| 78 | CD44 | E-cadherin | a | [[72](#_ENREF_72)] |

# S2 Table: gene function

|  | **Gene symbol** | **Gene function** |  |
| --- | --- | --- | --- |
| 1 | HIF-1α | hypoxia inducible factor-1 alpha  glycolysis | [[73](#_ENREF_73)] |
| 2 | P53 | Tumor suppressor gene | [[74](#_ENREF_74)] |
| 3 | c-myc | cell proliferation unlimited  cell apoptosis | [[56](#_ENREF_56)] |
| 4 | c-met | encodes hepatocyte growth factor receptor (c-met)  a potent gastric cancer drug target | [[75](#_ENREF_75)] |
| 5 | APC | Tumor suppressor gene  Gastric cancer and colon cancer | [[76](#_ENREF_76)] |
| 6 | TGF-β | Self-Sufficiency in Growth Signals  Metastasis | [[77](#_ENREF_77)] |
| 7 | TNF-α | Promote invasion and metastasis in gastric cancer  Tumor necrosis factor (TNF)-alpha | [[78](#_ENREF_78), [79](#_ENREF_79)] |
| 8 | ZEB | EMT related gene  Metastases | [[80](#_ENREF_80)] |
| 9 | VEGF | vascular endothelial growth factor  angiogenesis | [[81](#_ENREF_81)] |
| 10 | EGFR | proliferative signal  prognostic factors in gastric cancer | [[82](#_ENREF_82)] |
| 11 | hTERT | Unlimited replication  Telomerase related gene | [[83](#_ENREF_83)] |
| 12 | CDK2 | Evading growth suppressors | [[84](#_ENREF_84)] |
| 13 | IL-1β | wound healing  H. pylori infection | [[85](#_ENREF_85)] |
| 14 | P21 | a cyclin-dependent kinase inhibitor | [[86](#_ENREF_86)] |
| 15 | P27 | p27 is a cyclin-dependent kinase inhibitor and a putative tumor suppressor preventing progression of the cell cycle from G1 phase | [[87](#_ENREF_87)] |
| 16 | pRb | The retinoblastoma (Rb) pathway controls the G1-S checkpoint of the cell cycle | [[88](#_ENREF_88)] |
| 17 | E-cadherin | E-cadherin gene (CDH1) affect invasive/metastatic development of gastric cancer | [[89](#_ENREF_89)] |

S3 Table: expression value in each state. "0" represents low expression level, "1" represents high expression level. As the cancer is formed and developed during the life process, we defined the normal type as the wild type, and cancer type as the mutant type.

|  | **Gene symbol** | **in Cancer**  **(mutant)** | **In Normal (wild type)** | **literatures** | **State1**  **(IGC)** | **State2**  **(AG)** | **State3**  **(normal)** |
| --- | --- | --- | --- | --- | --- | --- | --- |
| 1 | p53 | 0 | 1 | [[90](#_ENREF_90)] | 1.1657 | 3.7559 | 5.5393 |
| 2 | c-myc | 1 | 0 | [[91](#_ENREF_91)] | 2.1585 | 2.0947 | 1.1030 |
| 3 | c-met | 1 | 0 | [[92](#_ENREF_92)] | 1.4067 | 1.4066 | 1.0329 |
| 4 | HIF-1α | 1 | 0 | [[93](#_ENREF_93)] | 2.6129 | 1.6446 | 1.0568 |
| 5 | APC | 0 | 1 | [[92](#_ENREF_92)] | 0.9934 | 1.0837 | 1.0976 |
| 6 | TGF-β | 1 | 0 | [[94](#_ENREF_94)] | 9.7215 | 7.4129 | 1.2324 |
| 7 | TNF-α | 1 | 0 | [[95](#_ENREF_95)] | 3.0727 | 1.8713 | 1.4008 |
| 8 | ZEB | 1 | 0 | [[96](#_ENREF_96)] | 1.0041 | 0.9770 | 0.8919 |
| 9 | EGFR | 1 | 0 | [[97](#_ENREF_97)] | 4.5628 | 4.3359 | 1.1318 |
| 10 | VEGF | 1 | 0 | [[81](#_ENREF_81)] | 5.3618 | 4.6415 | 1.1822 |
| 11 | hTERT | 1 | 0 | [[98](#_ENREF_98)] | 0.9961 | 0.7306 | 0.6390 |
| 12 | CDK2 | 1 | 0 | [[99](#_ENREF_99)] | 7.2469 | 5.0878 | 1.1677 |
| 13 | IL-1β | 1 | 0 | [[100](#_ENREF_100)] | 4.2739 | 4.2532 | 1.1561 |
| 14 | P21 | 1 | 0 | [[88](#_ENREF_88)] | 6.7991 | 6.7422 | 1.1829 |
| 15 | P27 | 1 | 0 | [[101](#_ENREF_101)] | 1.3737 | 1.3668 | 1.0164 |
| 16 | pRb | 1 | 0 | [[88](#_ENREF_88)] | 4.4360 | 1.6873 | 1.2225 |
| 17 | E-cadherin | 0 | 1 | [[102](#_ENREF_102)] | 1.1200 | 1.3923 | 2.8434 |

S4 Table: The weights (regulation strengths) of the network.

| 1.3434 | 1.8768 | 1.288 | 1.6942 | 1.1222 | 1.348 | 1.5829 | 1.5640 | 1.7445 | 1.9212 | 1.5349 | 2.286 | 1.8008 | 2.5644 | 1.9316 | 1.498 | 1.6208 |
| --- | --- | --- | --- | --- | --- | --- | --- | --- | --- | --- | --- | --- | --- | --- | --- | --- |
| 2.5092 | 1.7226 | 1.665 | 1.5163 | 1.8179 | 1.101 | 1.2868 | 1.0395 | 1.7429 | 1.0105 | 1.6000 | 1.785 | 1.9006 | 1.4359 | 1.8920 | 1.424 | 1.2326 |
| 1.3789 | 1.9728 | 1.797 | 1.7764 | 1.9669 | 1.932 | 1.4248 | 1.8770 | 1.3805 | 2.9197 | 1.7620 | 1.709 | 1.1862 | 1.2197 | 1.5486 | 1.284 | 1.7993 |
| 1.2198 | 1.2579 | 1.506 | 1.9626 | 1.1205 | 1.770 | 1.8998 | 1.0082 | 1.1054 | 1.7855 | 1.5000 | 1.788 | 1.2521 | 1.7887 | 1.8303 | 1.000 | 1.6471 |
| 1.0123 | 1.7824 | 1.702 | 1.7516 | 1.5059 | 1.469 | 1.0483 | 1.8608 | 1.8399 | 1.9275 | 1.3118 | 1.201 | 1.0446 | 1.3225 | 1.3890 | 1.755 | 1.9986 |
| 1.5331 | 1.6203 | 1.633 | 1.3023 | 1.3868 | 1.009 | 1.9358 | 1.5464 | 2.0788 | 2.3642 | 1.3000 | 1.864 | 2.2705 | 1.1103 | 1.1825 | 1.572 | 1.5943 |
| 1.7584 | 1.9793 | 1.433 | 1.4027 | 1.3444 | 1.810 | 2.4426 | 1.2616 | 1.2453 | 1.8870 | 1.2104 | 1.283 | 1.8869 | 1.0241 | 1.8394 | 2.533 | 1.9253 |
| 1.9022 | 1.1347 | 1.064 | 1.1481 | 1.1625 | 1.664 | 1.7211 | 1.7493 | 1.8164 | 1.2434 | 1.7504 | 1.082 | 1.4422 | 1.9494 | 1.7373 | 1.718 | 1.0500 |
| 1.5155 | 2.0135 | 1.491 | 1.7906 | 1.9112 | 1.075 | 2.0766 | 1.8702 | 1.9887 | 1.0480 | 1.8957 | 1.259 | 1.2603 | 1.3329 | 1.4073 | 1.739 | 1.1979 |
| 1.0360 | 1.7766 | 1.363 | 1.1375 | 1.3516 | 1.747 | 1.6607 | 1.3447 | 1.3325 | 1.2542 | 1.4022 | 1.067 | 1.0536 | 1.3278 | 1.3038 | 1.130 | 1.7275 |
| 1.3754 | 1.1128 | 1.898 | 1.7776 | 1.8302 | 1.721 | 2.4341 | 1.0147 | 1.0583 | 1.0819 | 1.1348 | 1.581 | 1.9324 | 1.1854 | 1.2287 | 1.290 | 1.8345 |
| 1.3909 | 1.1427 | 1.134 | 1.2701 | 1.2748 | 1.163 | 1.6195 | 1.0135 | 1.8188 | 1.6084 | 1.1714 | 1.851 | 1.9100 | 1.6683 | 1.8054 | 1.702 | 1.7883 |
| 1.7274 | 1.6360 | 1.878 | 1.7246 | 1.4999 | 1.787 | 1.7761 | 1.2635 | 1.1396 | 1.1064 | 1.2029 | 1.095 | 1.1094 | 1.0084 | 1.8890 | 1.113 | 1.3742 |
| 1.6226 | 1.5926 | 1.367 | 1.8168 | 1.9158 | 1.604 | 1.2770 | 1.7286 | 1.3132 | 1.4920 | 1.1951 | 1.167 | 1.1346 | 1.2761 | 1.7145 | 1.382 | 1.1835 |
| 1.9175 | 1.6524 | 1.332 | 1.2741 | 1.1150 | 1.854 | 1.9444 | 1.7886 | 1.2149 | 1.1500 | 1.2468 | 1.594 | 1.2502 | 2.0596 | 1.9815 | 1.076 | 1.6349 |
| 1.5590 | 1.5703 | 1.024 | 1.3690 | 1.1041 | 1.375 | 2.0247 | 1.6326 | 1.7102 | 1.2099 | 1.6561 | 1.778 | 1.7526 | 1.8119 | 1.8405 | 1.747 | 2.8744 |
| 1.026 | 1.1277 | 1.0576 | 1.8690 | 1.8744 | 1.1532 | 1.6178 | 1.8167 | 1.2075 | 1.6379 | 1.7256 | 1.0783 | 1.841 | 1.753 | 1.0380 | 2.2440 | 1.7873 |

S5 Table: The regulation type of the network. 1 represents the activation, -1 represents the repression.

| 0 | -1 | 0 | -1 | 1 | 1 | 0 | -1 | 1 | 0 | -1 | 1 | 0 | 1 | 0 | -1 | 0 |
| --- | --- | --- | --- | --- | --- | --- | --- | --- | --- | --- | --- | --- | --- | --- | --- | --- |
| 1 | -1 | 0 | 0 | 0 | 0 | 0 | 0 | 0 | 0 | 1 | 0 | 0 | 0 | 0 | -1 | 0 |
| 0 | 0 | -1 | 0 | 0 | 0 | 0 | 0 | 1 | 1 | 0 | 0 | 0 | 0 | 0 | 0 | 0 |
| 1 | 1 | 0 | 0 | 0 | 1 | 0 | 1 | 0 | 0 | -1 | 0 | 0 | 1 | 0 | 0 | 0 |
| 1 | -1 | 0 | 0 | -1 | 0 | 0 | 0 | 0 | 0 | 0 | 0 | 0 | 0 | 0 | 1 | 0 |
| 1 | 0 | 0 | 0 | 0 | 1 | 1 | 1 | 1 | 1 | -1 | -1 | 1 | 1 | 0 | 0 | 0 |
| 1 | 1 | 1 | 1 | 0 | 1 | 1 | 0 | 1 | 0 | 0 | 1 | 1 | 0 | 0 | 1 | 0 |
| 0 | 0 | 0 | 0 | 0 | 1 | 0 | -1 | 0 | 0 | 0 | 1 | 0 | 0 | 0 | 0 | 0 |
| 1 | 1 | 0 | 0 | 0 | 0 | 1 | 0 | 0 | 0 | 0 | 0 | 0 | 1 | 1 | 0 | 0 |
| 0 | 0 | 0 | 1 | 0 | 0 | 0 | 0 | 0 | 0 | 0 | 0 | 0 | 1 | 0 | 1 | 0 |
| 0 | 0 | 0 | 0 | 0 | 0 | 1 | 0 | 0 | 1 | 0 | 0 | 0 | 0 | 0 | 0 | 0 |
| 0 | 1 | 0 | 0 | 0 | 0 | 0 | 0 | 0 | 0 | 0 | 1 | 0 | 0 | 0 | 0 | 0 |
| 0 | 0 | 0 | 0 | 0 | 1 | 1 | 0 | 0 | 1 | 0 | 0 | 0 | 0 | 0 | 1 | 0 |
| 0 | -1 | 0 | 0 | 0 | 1 | 0 | 0 | 0 | 1 | 0 | 0 | 0 | 1 | 0 | 0 | 0 |
| 0 | 0 | 0 | 1 | 0 | 0 | 0 | 0 | 0 | 0 | 0 | 0 | 0 | 1 | 0 | 0 | 1 |
| -1 | 0 | 0 | 1 | 0 | 1 | 1 | 0 | 0 | 1 | 0 | 1 | 0 | 0 | 0 | 1 | 1 |
| 0 | 0 | 0 | 0 | 0 | 0 | 0 | 0 | 0 | 0 | 0 | 0 | 0 | 0 | 0 | 1 | 0 |

1. **The cross-correlation of forward and backward in time**

In Fig.S1, we show the simulated stochastic trajectories of the genes CDH1 and TGF-β as an example. Fig.S1 displays the cross-correlation functions between CDH1 and TGF-β forward and backward in time. The black line represents the forward cross-correlation, and the blue line represents the backward cross-correlation. Fig.S1(B) displays the difference between the forward and backward cross-correlation functions in time of the two genes. The black and blue lines (forward and backward) are separated, indicating time irreversibility. When the flux is zero, the forward and backward cross-correlation functions are equal. However, in a non-equilibrium system where the flux is nonzero, the forward and backward cross-correlation functions differ and exhibit time irreversibility.

In summary, by calculating the differences between forward and backward cross-correlation functions, we can quantify nonequilibrium dynamics in cancer systems without directly measuring the EPR or mean flux. This method provides a useful tool for identifying early warning signals for critical phase transitions in cancer systems.

Figure S1.The simulated trajectories of the gene expressions of CDH1 and TGF-β. (A) The time forward (black) and time backward (blue) cross correlation functions between the gene of CDH1 and TGF-β. (B) The difference of the forward and backward cross correlation functions.

**References**

[1]Hou, L., et al., *Effect of hyperlipidemia on the expression of circadian genes in apolipoprotein E knock-out atherosclerotic mice.* Lipids in Health and Disease, 2009. **8**.

[2]Yang, J., et al., *Small-Molecule Activation of p53 Blocks Hypoxia-Inducible Factor 1 alpha and Vascular Endothelial Growth Factor Expression In Vivo and Leads to Tumor Cell Apoptosis in Normoxia and Hypoxia.* Molecular and Cellular Biology, 2009. **29**(8): p. 2243-2253.

[3]Kim, T., et al., *p53 regulates epithelial-mesenchymal transition through microRNAs targeting ZEB1 and ZEB2.* Journal of Experimental Medicine, 2011. **208**(5): p. 875-883.

[4]Li, P.X., et al., *Placental transforming growth factor-beta is a downstream mediator of the growth arrest and apoptotic response of tumor cells to DNA damage and p53 overexpression.* Journal of Biological Chemistry, 2000. **275**(26): p. 20127-20135.

[5]Jaiswal, A.S., et al., *N-Methyl-N '-nitro-N-nitrosoguanidine-induced senescence-like growth arrest in colon cancer cells is associated with loss of adenomatous polyposis coli protein, microtubule organization, and telomeric DNA.* Molecular Cancer, 2004. **3**.

[6]Klanrit, P., et al., *PML involvement in the p73-mediated E1A-induced suppression of EGFR and induction of apoptosis in head and neck cancers.* Oncogene, 2009. **28**(39): p. 3499-3512.

[7]Sanduja, S., V. Kaza, and D.A. Dixon, *The mRNA decay factor tristetraprolin (TTP) induces senescence in human papillomavirus-transformed cervical cancer cells by targeting E6-AP ubiquitin ligase.* Cancer Research, 2010. **70**.

[8]Gil-Gomez, G., A. Berns, and H.J.M. Brady, *A link between cell cycle and cell death: Bax and Bcl-2 modulate Cdk2 activation during thymocyte apoptosis.* Embo Journal, 1998. **17**(24): p. 7209-7218.

[9]Sa, G. and T. Das, *Anti cancer effects of curcumin: cycle of life and death.* Cell Division, 2008. **3**.

[10]Abe, M., et al., *Mechanisms of confluence-dependent expression of CD26 in colon cancer cell lines.* Bmc Cancer, 2011. **11**.

[11]Chen, J., C. Lin, and Y. Deng, *The effects of RA538 and antisense c-myc on cervical cancer cell lines with high expression of bcl-2 gene.* Zhonghua zhong liu za zhi [Chinese journal of oncology], 2000. **22**(4): p. 279-82.

[12]Skvortzov, D.A., et al., *The Regulation of Telomerase in Oncogenesis.* Acta Naturae, 2009. **1**(1): p. 51-67.

[13]Mueller, K.L., et al., *EGFR/Met association regulates EGFR TKI resistance in breast cancer.* Journal of molecular signaling, 2010. **5**: p. 8-8.

[14]Grepin, R. and G. Pages, *Molecular mechanisms of resistance to tumour anti-angiogenic strategies.* Journal of oncology, 2010. **2010**: p. 835680-835680.

[15]Gao, X. and D. Xing, *Molecular mechanisms of cell proliferation induced by low power laser irradiation.* Journal of Biomedical Science, 2009. **16**.

[16]Kim, M.K., et al., *Clinical significance of HIF-2 alpha immunostaining area in radioresistant cervical cancer.* Journal of Gynecologic Oncology, 2011. **22**(1): p. 44-48.

[17]Gorospe, M., et al., *Post-transcriptional control of the hypoxic response by RNA-binding proteins and microRNAs.* Frontiers in Molecular Neuroscience, 2011. **4**.

[18]Foubert, E., B. De Craene, and G. Berx, *Key signalling nodes in mammary gland development and cancer. The Snail1-Twist1 conspiracy in malignant breast cancer progression.* Breast Cancer Research, 2010. **12**(3).

[19]Yu, R.M., et al., *Hypoxia induces telomerase reverse transcriptase (TERT) gene expression in non-tumor fish tissues in vivo: the marine medaka (Oryzias melastigma) model.* Bmc Molecular Biology, 2006. **7**.

[20]Iervolino, A., et al., *Bcl-2 overexpression in human melanoma cells increases angiogenesis through VEGF mRNA stabilization and HIF-1-mediated transcriptional activity.* Faseb Journal, 2002. **16**(9): p. 1453-+.

[21]Otsuki, Y., *Apoptosis in human endometrium: apoptotic detection methods and signaling.* Medical electron microscopy : official journal of the Clinical Electron Microscopy Society of Japan, 2001. **34**(3): p. 166-73.

[22]Beham, A., et al., *Bcl-2 inhibits p53-induced apoptosis after genotoxic damage by inhibitors of nuclear import of p53.* Langenbecks Archiv fur Chirurgie. Supplement. Kongressband. Deutsche Gesellschaft fur Chirurgie. Kongress, 1998. **115**(Suppl I): p. 113-7.

[23]Rosenwald, I.B., et al., *Upregulation of protein synthesis initiation factor eIF-4E is an early event during colon carcinogenesis.* Oncogene, 1999. **18**(15): p. 2507-2517.

[24]Macias, W.L., et al., *New insights into the protein C pathway: potential implications For the biological activities of drotrecogin alfa (activated).* Critical Care, 2005. **9**: p. S38-S45.

[25]Liu, J., et al., *Siah-1 mediates a novel beta-catenin degradation pathway linking p53 to the adenomatous polyposis coli protein.* Molecular Cell, 2001. **7**(5): p. 927-936.

[26]Park, K.H., et al., *Downregulation of the anaphase-promoting complex (APC)7 in invasive ductal carcinomas of the breast and its clinicopathologic relationships.* Breast Cancer Research, 2005. **7**(2): p. R238-R247.

[27]Patel, P., et al., *Transforming growth factor beta induces mesangial cell apoptosis through NO- and p53-dependent and -independent pathways.* Journal of Investigative Medicine, 2000. **48**(6): p. 403-410.

[28]Oh, J.W., L.M. Schwiebert, and E.N. Benveniste, *Cytokine regulation of CC and CXC chemokine expression by human astrocytes.* Journal of Neurovirology, 1999. **5**(1): p. 82-94.

[29]Ungefroren, H., et al., *Interaction of tumor cells with the microenvironment.* Cell Communication and Signaling, 2011. **9**.

[30]Janssens, K., et al., *Transforming growth factor-beta 1 mutations in Camurati-Engelmann disease lead to increased signaling by altering either activation or secretion of the mutant protein.* Journal of Biological Chemistry, 2003. **278**(9): p. 7718-7724.

[31]Cho, J.-H., et al., *Systems biology of interstitial lung diseases: integration of mRNA and microRNA expression changes.* Bmc Medical Genomics, 2011. **4**.

[32]Gille, J., R.A. Swerlick, and S.W. Caughman, *Transforming growth factor-alpha-induced transcriptional activation of the vascular permeability factor (VPF/VEGF) gene requires AP-2-dependent DNA binding and transactivation.* Embo Journal, 1997. **16**(4): p. 750-759.

[33]Elkak, A.E., et al., *Is telomerase reactivation associated with the down-regulation of TGF beta receptor-II expression in human breast cancer?* Cancer Cell International, 2003. **3**.

[34]Kim, S., A.J. Schein, and J.A. Nadel, *E-cadherin promotes EGFR-mediated cell differentiation and MUC5AC mucin expression in cultured human airway epithelial cells.* American Journal of Physiology-Lung Cellular and Molecular Physiology, 2005. **289**(6): p. L1049-L1060.

[35]Donovan, J. and J. Slingerland, *Transforming growth factor-beta and breast cancer - Cell cycle arrest by transforming growth factor-beta and its disruption in cancer.* Breast Cancer Research, 2000. **2**(2): p. 116-124.

[36]Xu, H., et al., *Anti-malarial agent artesunate inhibits TNF-alpha-induced production of proinflammatory cytokines via inhibition of NF-kappa B and PI3 kinase/Akt signal pathway in human rheumatoid arthritis fibroblast-like synoviocytes.* Rheumatology, 2007. **46**(6): p. 920-926.

[37]Dimmeler, S., et al., *Dephosphorylation targets Bcl-2 for ubiquitin-dependent degradation: A link between the apoptosome and the proteasome pathway.* Journal of Experimental Medicine, 1999. **189**(11): p. 1815-1822.

[38]Aspalter, R.M., M.M. Eibl, and H.M. Wolf, *Regulation of TCR-mediated T cell activation by TNF-RII.* Journal of Leukocyte Biology, 2003. **74**(4): p. 572-582.

[39]van Uden, P., N.S. Kenneth, and S. Rocha, *Regulation of hypoxia-inducible factor-1 alpha by NF-kappa B.* Biochemical Journal, 2008. **412**: p. 477-484.

[40]Chen, Q.Y., M.C. DeFrances, and R. Zarnegar, *Induction of met proto-oncogene (Hepatocyte growth factor receptor) expression during human monocyte-macrophage differentiation.* Cell Growth & Differentiation, 1996. **7**(6): p. 821-832.

[41]Lopez-Marure, R., et al., *Ceramide mimics tumour necrosis factor-alpha in the induction of cell cycle arrest in endothelial cells - Induction of the tumour suppressor p53 with decrease in retinoblastoma/protein levels.* European Journal of Biochemistry, 2000. **267**(14): p. 4325-4333.

[42]Hayashi, T., et al., *Influence of alpha-tumor necrosis factor and beta-interleukin-1 on production of angiogenetic factors and thymidine phosphorylase activity in immortalized human decidual fibroblasts in vitro.* Journal of Obstetrics and Gynaecology Research, 2006. **32**(1): p. 15-22.

[43]Chakraborti, S. and T. Chakraborti, *Oxidant-mediated activation of mitogen-activated protein kinases and nuclear transcription factors in the cardiovascular system: A brief overview.* Cellular Signalling, 1998. **10**(10): p. 675-683.

[44]Lee, I.T., et al., *TNF-alpha Induces Matrix Metalloproteinase-9 Expression in A549 Cells: Role of TNFR1/TRAF2/PKC alpha-Dependent Signaling Pathways.* Journal of Cellular Physiology, 2010. **224**(2): p. 454-464.

[45]Akiyama, M., et al., *Nuclear factor-kappa B p65 mediates tumor necrosis factor alpha-induced nuclear translocation of telomerase reverse transcriptase protein.* Cancer Research, 2003. **63**(1): p. 18-21.

[46]Tsuji, Y., et al., *SELECTIVE LOSS OF CDC2 AND CDK2 INDUCTION BY TUMOR-NECROSIS-FACTOR-ALPHA IN SENESCENT HUMAN-DIPLOID FIBROBLASTS.* Experimental Cell Research, 1993. **209**(2): p. 175-182.

[47]Sass, S., et al., *MicroRNAs coordinately regulate protein complexes.* Bmc Systems Biology, 2011. **5**.

[48]Gauger, K., et al., *SFRP1 reduction results in an increased sensitivity to TGF-beta signaling.* Cancer Research, 2011. **71**.

[49]Hu, F., et al., *DeltaEF1 promotes breast cancer cell proliferation through down-regulating p21 expression.* Cancer Research, 2010. **70**.

[50]Habib, A.A., et al., *The epidermal growth factor receptor engages receptor interacting protein and nuclear factor-kappa B (NF-kappa B)-inducing kinase to activate NF-kappa B - Identification of a novel receptor-tyrosine kinase signalosome.* Journal of Biological Chemistry, 2001. **276**(12): p. 8865-8874.

[51]Yian, X., et al., *Mechanism of antisense epidermal growht factor receptor cDNA in growth suppression of glioblastomas cells.* Chinese Journal of Pathology, 2003. **32**(3): p. 242-246.

[52]Huo, Q., *A Functional Nuclear Epidermal Growth Factor Receptor, Src and Stat3 Heteromeric Complex in Pancreatic Cancer Cells (vol 14, e0212884, 2019).* Plos One, 2019. **14**(4).

[53]Zheng, L.-H., et al., *Antitumor Peptides from Marine Organisms.* Marine Drugs, 2011. **9**(10): p. 1840-1859.

[54]Zhou, L., et al., *Telomerase reverse transcriptase activates the expression of vascular endothelial growth factor independent of telomerase activity.* Biochemical and Biophysical Research Communications, 2009. **386**(4): p. 739-743.

[55]Stampfer, M.R., et al., *Expression of the telomerase catalytic subunit, hTERT, induces resistance to transforming growth factor beta growth inhibition in p16(INK4A)(-) human mammary epithelial cells.* Proceedings of the National Academy of Sciences of the United States of America, 2001. **98**(8): p. 4498-4503.

[56]Zou, X.M., et al., *Induction of c-myc transcription by the v-Abl tyrosine kinase requires Ras, Raf1, and cyclin-dependent kinases.* Genes & Development, 1997. **11**(5): p. 654-662.

[57]Perkins, N.D., et al., *Regulation of NF-kappa B by cyclin-dependent kinases associated with the p300 coactivator.* Science, 1997. **275**(5299): p. 523-527.

[58]Turzanski, J., et al., *Interleukin-1 beta maintains an apoptosis-resistant phenotype in the blast cells of acute myeloid leukaemia via multiple pathways.* Leukemia, 2004. **18**(10): p. 1662-1670.

[59]Okigaki, M., et al., *Mechanism for IL-1 beta-mediated neovascularization unmasked by IL-1 beta knock-out mice.* Circulation, 2005. **112**(17): p. U299-U299.

[60]Isumi, Y., et al., *Adrenomedullin suppresses interleukin-1 beta-induced tumor necrosis factor-alpha production in Swiss 3T3 cells.* Febs Letters, 1999. **463**(1-2): p. 110-114.

[61]Lenos, K. and A.G. Jochemsen, *Functions of MDMX in the Modulation of the p53-Response.* Journal of Biomedicine and Biotechnology, 2011.

[62]Sabin, R.J. and R.M. Anderson, *Cellular Senescence - its role in cancer and the response to ionizing radiation.* Genome integrity, 2011. **2**(1): p. 7.

[63]Zhang, G., et al., *Multiple cyclin kinase inhibitors promote bile acid-induced apoptosis and autophagy in primary hepatocytes via p53-CD95-dependent signaling (vol 283, pg 24343, 2008).* Journal of Biological Chemistry, 2016. **291**(39): p. 20823-20823.

[64]Unahara, Y., et al., *Cellular thiol status-dependent inhibition of tumor cell growth via modulation of p27<SUP>kip1</SUP> stop translocation and retinoblastoma protein phosphorylation by 1′-acetoxychavicol acetate.* Amino Acids, 2007. **33**(3): p. 469-476.

[65]Shankland, S.J., et al., *Differential expression of cyclin-dependent kinase inhibitors in human glomerular disease: Role in podocyte proliferation and maturation.* Kidney International, 2000. **58**(2): p. 674-683.

[66]Kim, S.J., et al., *RETINOBLASTOMA GENE-PRODUCT ACTIVATES EXPRESSION OF THE HUMAN TGF-BETA-2 GENE THROUGH TRANSCRIPTION FACTOR ATF-2.* Nature, 1992. **358**(6384): p. 331-334.

[67]Wu, J.B., et al., *Progestin-dependent induction of vascular endothelial growth factor in human breast cancer cells: Preferential regulation by progesterone receptor B.* Cancer Research, 2004. **64**(6): p. 2238-2244.

[68]Salcedo, M., et al., *The retinoblastoma gene product negatively regulates cellular or viral oncogene promoters in vivo.* Archives of Medical Research, 1995. **26**: p. S157-S162.

[69]Imamichi, Y., et al., *Collagen type I-induced Smad-interacting protein 1 expression downregulates E-cadherin in pancreatic cancer.* Oncogene, 2007. **26**(16): p. 2381-2385.

[70]Qian, X.L., et al., *E-cadherin-mediated adhesion inhibits ligand-dependent activation of diverse receptor tyrosine kinases.* Embo Journal, 2004. **23**(8): p. 1739-1748.

[71]Gamboa-Dominguez, A., et al., *Prognostic significance of p21<SUP>WAF1/CIP1</SUP>, p27<SUP>Kip1</SUP>, p53 and E-cadherin expression in gastric cancer.* Journal of Clinical Pathology, 2007. **60**(7): p. 756-761.

[72]Le Bras, G.F., et al., *CD44 Upregulation in E-Cadherin-Negative Esophageal Cancers Results in Cell Invasion.* Plos One, 2011. **6**(11).

[73]Wang, T., et al., *HIF1 alpha-Induced Glycolysis Metabolism Is Essential to the Activation of Inflammatory Macrophages.* Mediators of Inflammation, 2017.

[74]DeVries, E.M.G., et al., *Database of mutations in the p53 and APC tumor suppressor genes designed to facilitate molecular epidemiological analyses.* Human Mutation, 1996. **7**(3): p. 202-213.

[75]Gymnopoulos, M., et al., *TR1801-ADC: a highly potent cMet antibody-drug conjugate with high activity in patient-derived xenograft models of solid tumors.* Molecular Oncology, 2020. **14**(1): p. 54-68.

[76]Jiang, Y., J. Przybyszewski, and D.F. Birt, *Resveratrol inhibits cell growth and induces cell cycle arrest in human colon cancer cells independently of tumor suppressor genes p53 and APC.* Cancer Epidemiology Biomarkers & Prevention, 2004. **13**(11): p. 1912S-1912S.

[77]Sarkar, A., et al., *TGF beta mediated LINC00273 upregulation sponges mir200a-3p and promotes invasion and metastasis by activating ZEB1.* Journal of Cellular Physiology.

[78]Cui, X., et al., *Cytokine TNF-alpha promotes invasion and metastasis of gastric cancer by down-regulating Pentraxin3.* Journal of Cancer, 2020. **11**(7): p. 1800-1807.

[79]Siregar, G.A., S. Halim, and V.R. Sitepu, *Serum TNF-a, IL-8, VEGF levels in Helicobacter pylori infection and their association with degree of gastritis.* Acta medica Indonesiana, 2015. **47**(2): p. 120-6.

[80]Wellner, U., et al., *The EMT-activator ZEB1 promotes tumorigenicity by repressing stemness-inhibiting microRNAs.* Nature Cell Biology, 2009. **11**(12): p. 1487-U236.

[81]Lieto, E., et al., *Expression of vascular endothelial growth factor (VEGF) and epidermal growth factor receptor (EGFR) is an independent prognostic indicator of worse outcome in gastric cancer patients.* Annals of Surgical Oncology, 2008. **15**(1): p. 69-79.

[82]Parker, M.I., et al., *Proliferative signaling by ERBB proteins and RAF/MEK/ERK effectors in polycystic kidney disease.* Cellular Signalling, 2020. **67**.

[83]Ohira, T., et al., *PITX1 protein interacts with ZCCHC10 to regulate hTERT mRNA transcription.* Plos One, 2019. **14**(8).

[84]Strobeck, M.W., et al., *Restoration of retinoblastoma mediated signaling to Cdk2 results in cell cycle arrest.* Oncogene, 2000. **19**(15): p. 1857-1867.

[85]Bakulina, N.V., et al., *Efficacy of H. pylori eradication depending on genetic polymorphism of CYP2C19, MDR1 and IL-1 beta.* Terapevticheskii Arkhiv, 2019. **91**(8): p. 34-40.

[86]Yang, M., et al., *New substituted heterocyclic compounds useful as potent p21-activated kinase 4 inhibitor in medicament to treat e.g. breast cancer, melanoma, bladder cancer and kidney cancer, neurodegenerative disease and immune system disease*. CHENGDU HYPERWAY PHARM CO LTD (CHEN-Non-standard) SHENZHEN HYPERWAY PHARM CO LTD (SHEN-Non-standard) SHENZHEN HAIBOWEI PHARM CO LTD (SHEN-Non-standard) HYPERWAY PHARM CO LTD (HYPE-Non-standard).

[87]Wiksten, J.P., et al., *The prognostic value of p27 in gastric cancer.* Oncology, 2002. **63**(2): p. 180-184.

[88]Feakins, R.M., et al., *Abnormal expression of pRb, p16, and cyclin D1 in gastric adenocarcinoma and its lymph node metastases: Relationship with pathological features and survival.* Human Pathology, 2003. **34**(12): p. 1276-1282.

[89]Zhan, Z., et al., *<i>CDH1</i> gene polymorphisms, plasma CDH1 levels and risk of gastric cancer in a Chinese population.* Molecular Biology Reports, 2012. **39**(8): p. 8107-8113.

[90]Yang, P., et al., *The impact of p53 in predicting clinical outcome of breast cancer patients with visceral metastasis.* Scientific Reports, 2013. **3**: p. 6.

[91]Calcagno, D.Q., et al., *MYC, FBXW7 and TP53 copy number variation and expression in Gastric Cancer.* Bmc Gastroenterology, 2013. **13**.

[92]Wang, J.Y., et al., *Alterations of APC, c-met, and p53 genes in tumor tissue and serum of patients with gastric cancers.* Journal of Surgical Research, 2004. **120**(2): p. 242-248.

[93]Rohwer, N., et al., *HIF-1 alpha determines the metastatic potential of gastric cancer cells.* British Journal of Cancer, 2009. **100**(5): p. 772-781.

[94]Fu, H., et al., *TGF-beta promotes invasion and metastasis of gastric cancer cells by increasing fascin1 expression via ERK and JNK signal pathways.* Acta Biochimica Et Biophysica Sinica, 2009. **41**(8): p. 648-656.

[95]Izutani, R., et al., *Enhanced expression of manganese superoxide dismutase mRNA and increased TNF alpha mRNA expression by gastric mucosa in gastric cancer.* World Journal of Surgery, 1996. **20**(2): p. 228-233.

[96]Chen, B., et al., *Prognostic value of ZEB-1 in solid tumors: a meta-analysis.* Bmc Cancer, 2019. **19**.

[97]Terashima, M., et al., *Impact of Expression of Human Epidermal Growth Factor Receptors EGFR and ERBB2 on Survival in Stage II/III Gastric Cancer.* Clinical Cancer Research, 2012. **18**(21): p. 5992-6000.

[98]Yao, X.X., L. Yin, and Z.C. Sun, *The expression of hTERT mRNA and cellular immunity in gastric cancer and precancerosis.* World Journal of Gastroenterology, 2002. **8**(4): p. 586-590.

[99]Nakayama, S., et al., *Prediction of paclitaxel sensitivity by CDK1 and CDK2 activity in human breast cancer cells.* Breast Cancer Research, 2009. **11**(1): p. 10.

[100]Li, S., et al., *IL-1 beta mediates MCP-1 induction by Wnt5a in gastric cancer cells.* Bmc Cancer, 2014. **14**.

[101]Bayrak, M., et al., *Prognostic significance of c-erbB2 overexpression in patients with metastatic gastric cancer.* Clinical & Translational Oncology, 2013. **15**(4): p. 307-312.

[102]Berx, G., et al., *Mutations of the human E-cadherin (CDH1) gene.* Human Mutation, 1998. **12**(4): p. 226-237.
